# Supplementary material for: Myeloid Cell and Transcriptome Signatures Associated With Inflammation Resolution in a Model of Self-Limiting Acute Brain Inflammation
Source: Front Immunol. 2019 May 17;10:1048. doi: 10.3389/fimmu.2019.01048 (PMC6533855; doi:10.3389/fimmu.2019.01048)
Supplement: Supplementary file 5 [file Data_Sheet_1.PDF]

Supplementary Figure 1

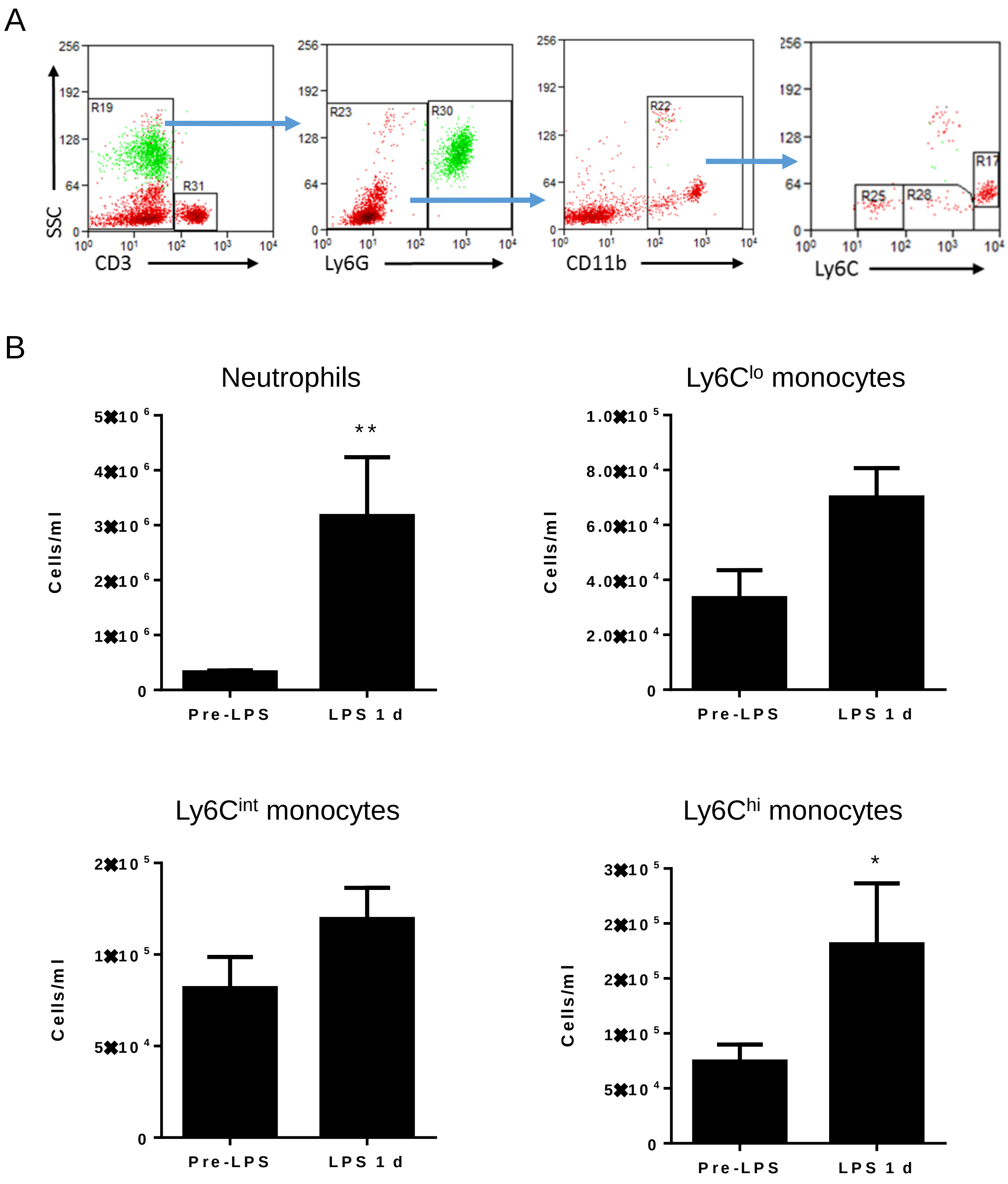

Supplementary Figure 1. (A) Flow cytometry gating scheme to identify blood myeloid cell populations. $CD3^-$ cells were selected and then neutrophils defined as $Ly6G^+SSC^{hi}$. $Ly6G^-CD11b^+$ cells were analysed for Ly6C – three distinct monocyte populations were identified based on intensity of Ly6C expression. (B) Numbers of blood myeloid cell populations in venous blood samples collected before and after intracerebral LPS injection. $*P < 0.05$, $**P < 0.01$; Student's t test. N = 4-6 mice per group.
